# Subliminal Emotional Words Impact Syntactic Processing: Evidence from Performance and Event-Related Brain Potentials

**DOI:** 10.3389/fnhum.2017.00192

**Published:** 2017-04-25

**Authors:** Laura Jiménez-Ortega, Javier Espuny, Pilar Herreros de Tejada, Carolina Vargas-Rivero, Manuel Martín-Loeches

**Affiliations:** ^1^Centre for Human Evolution and Behaviour, Instituto de Salud Carlos III, Complutense University of Madrid (UCM-ISCIII)Madrid, Spain; ^2^Psychobiology Department, Complutense University of MadridMadrid, Spain

**Keywords:** language comprehension, unconscious processing, emotional effects, subliminal presentation, syntactic processing, LAN, P600

## Abstract

Recent studies demonstrate that syntactic processing can be affected by emotional information and that subliminal emotional information can also affect cognitive processes. In this study, we explore whether unconscious emotional information may also impact syntactic processing. In an Event-Related brain Potential (ERP) study, positive, neutral and negative subliminal adjectives were inserted within neutral sentences, just before the presentation of the supraliminal adjective. They could either be correct (50%) or contain a morphosyntactic violation (number or gender disagreements). Larger error rates were observed for incorrect sentences than for correct ones, in contrast to most studies using supraliminal information. Strikingly, emotional adjectives affected the conscious syntactic processing of sentences containing morphosyntactic anomalies. The neutral condition elicited left anterior negativity (LAN) followed by a P600 component. However, a lack of anterior negativity and an early P600 onset for the negative condition were found, probably as a result of the negative subliminal correct adjective capturing early syntactic resources. Positive masked adjectives in turn prompted an N400 component in response to morphosyntactic violations, probably reflecting the induction of a heuristic processing mode involving access to lexico-semantic information to solve agreement anomalies. Our results add to recent evidence on the impact of emotional information on syntactic processing, while showing that this can occur even when the reader is unaware of the emotional stimuli.

## Introduction

We are surrounded by endless emotional stimulation. Indeed, detecting and processing emotional information has an enormous adaptive value. This is so to such an extent that, during recent decades, it has been observed that emotions interact with almost all cognitive domains investigated, such as planning, attention, memory, decision making, or language (Ashby et al., 1999; Mitchell and Phillips, 2007; Pessoa, 2008; Vissers et al., 2010; Jiménez-Ortega et al., 2012; Martín-Loeches et al., 2012). Overall, recent views on brain function and anatomy pose the foundations of rich, extensive interweaving between emotion and cognition, which exhibit highly overlapping networks (Pessoa, 2008, 2012, 2015b, 2016).

A large amount of emotional information is often unconsciously processed. A growing number of experiments demonstrate that unconscious stimuli impact cognitive processes at several levels (for reviews see Dehaene et al., 2006; Van den Bussche et al., 2009; Kiefer et al., 2011). Remarkably, even subliminal emotional words can trigger long-lasting cerebral processes. For instance, Naccache et al. (2005) found by means of intracranial recordings that threatening subliminal words modulate the activity of the amygdala at long latencies. More recently, Gibbons (2009), by means of Event-Related brain Potentials (ERP), observed the effects of subliminal emotional words on preference judgments regarding subsequent target stimuli such as paintings and portraits. Targets preceded by positive arousing primes were preferred to targets preceded by negative and non-arousing positive primes. Overall, the impact of subliminal words on cognitive processing appears to be supported.

Another relevant area of interest is how emotions affect language comprehension. Although the modulation of language processing by emotions has often been investigated by using isolated words as targets, or focusing on semantic sentence processing (Kiefer et al., 2007), recent studies have also explored emotional effects on the syntactic processing of a sentence (Vissers et al., 2010; Jiménez-Ortega et al., 2012; Martín-Loeches et al., 2012; Hinojosa et al., 2014; Verhees et al., 2015). The effects of emotional words on syntactic processing as reflected in ERP data seem to have been proved, in spite of the traditional view that syntax is an encapsulated process (Fodor, 1983; Hauser et al., 2002). In the present article, we wanted to go further by exploring whether subliminally presented emotional words may impact syntactic processes.

A suitable way to study emotional impact on syntax has been to check how emotional information modulates syntactic ERP components. Traditionally, two functionally distinct ERP components related to syntax have been commonly reported; anterior negativities (ANs) and P600 (or late positive complex (LPC)). While the P600 component is also related to semantic and integration processes, ANs are triggered by syntactic anomalies (e.g., Rothermich et al., 2010; Steinhauer and Drury, 2012; Bohn et al., 2013; Magne et al., 2016). Particularly, ANs appear in response to grammatical anomalies, such as morphosyntactic violations (a violation of the formal relation between two linguistic forms; e.g., a number disagreement between noun and verb), between 300 ms and 500 ms after the stimulus onset over frontal electrodes. It is typically left-sided, and this is the reason why it is also known as left anterior negativity (LAN), though fronto-central distributions are not rare. They seem to reflect highly automatic first parsing processes, the detection of morphosyntactic mismatches, the difficulty of processing correct but rare grammatical structures, or the inability to assign the incoming word to the current phrase structure (Friederici, 1995; Rösler et al., 1998; Hahne and Friederici, 1999; Hagoort, 2003b). ANs seem also to reflect some aspects of working memory operations (King and Kutas, 1995; Weckerly and Kutas, 1999; Martín-Loeches et al., 2005; Makuuchi et al., 2009). In addition, ANs have also been reported as a response to violations of rhythm-based expectations (e.g., Böcker et al., 1999; Schmidt-Kassow and Kotz, 2009; Bohn et al., 2013; Magne et al., 2016). The P600 reaction to syntactic anomalies appears between 600 ms and 900 ms after the onset of the anomaly over centro-parietal electrodes (Friederici et al., 2004). Traditionally, it is believed that the P600 component reflects the costs of repair and revision of structural mismatches and/or integration processes between semantic and syntactic information (Kuperberg et al., 2000; Friederici et al., 2002; Martín-Loeches et al., 2006). Most recently, it has been suggested that it may also reflect the integration processes of conscious and unconscious linguistic information (Jiménez-Ortega et al., 2014).

In addition, two emotion-related ERP components have been typically reported in relation to emotional words: early posterior negativity (EPN; Junghöfer et al., 2001; Herbert et al., 2006, 2008; Kissler et al., 2009; Schacht and Sommer, 2009a) and the LPC (Fischler and Bradley, 2006; Schacht and Sommer, 2009b). EPN is a temporo-occipital negativity around 200 ms post-stimulus, which reflects voluntary orientation and attention, in which the task-relevant stimuli are selected for further, more elaborate processing (Potts et al., 2008). The LPC component for emotional words is observed at around 500 ms after the stimulus onset. It reflects elaborate emotional processing, and has been interpreted as the increment of intrinsic relevance, motivational significance and arousal value of the emotional stimuli in comparison with neutral stimuli (Schupp et al., 2000, 2013; Schacht and Sommer, 2009a).

As mentioned above, contrary to classical models of syntax as an encapsulated process, recent evidence suggests that emotional information modulates syntactic processing, as reflected in the ANs and the P600 syntactic components triggered by morphosyntactic anomalies. The AN response to morphosyntactic violations has been seen to be affected by emotional paragraphs; in this regard, while it was not visible in the neutral condition, it was triggered in the negative and positive conditions (Jiménez-Ortega et al., 2012). In the study by Martín-Loeches et al. (2012), the emotional information was part of the sentence being processed. They tested emotional effects on language comprehension and particularly on syntactic processing by presenting emotional adjectives, which could be syntactically correct or incorrect (number-agreement violations) with respect to the ongoing sentence. They observed that the amplitude of the AN increased for negative adjectives containing morphosyntactic violations, while it decreased for positive adjectives, in comparison with neutral ones. The results of the study by Hinojosa et al. (2014) follow a similar line. On the other hand, P600 modulations have been reported when presenting happy and sad film clips preceding subject-verb agreement violations (Vissers et al., 2010; Verhees et al., 2015). By using a similar procedure, Van Berkum et al. (2013) observed a slightly earlier onset of P600 effects in the happy mood condition as against the sad one. Finally, using emotionally-laden words with gender disagreements in sentences (Díaz-Lago et al., 2015) have reported late modulations of the P600 component as a function of emotionality.

As can be appreciated, the effects of emotional (i.e., lexico-semantic) information on syntactic processing have been reported both in the AN and the P600 (both syntactic) components of the ERP. If AN has been affected, this means that the lexico-semantic information conveyed by emotional words or texts is able to impact syntactic processing at its early and presumably automatic stages (Hasting and Kotz, 2008; Batterink and Neville, 2013; Jiménez-Ortega et al., 2014; Lucchese et al., 2017). These findings therefore not only challenge modular and encapsulated, sequential models of language processing (e.g., Fodor, 1983; Ullman, 2001, 2004; Friederici, 2002, 2006; Hauser et al., 2002; Hagoort, 2003a) but further support fully interactive views of language, that is, that interactions between lexico-semantic and syntactic information can occur from the very beginning (e.g., MacDonald et al., 1994; Novick et al., 2003; Kuperberg, 2007; Pickering and Garrod, 2013), even within the first 200 ms after the onset of an anomaly (e.g., Lucchese et al., 2017).

The present study aims to go further relative to previous literature and explore the possibility that early automatic syntax-related modulations (as reflected in ANs) can also be affected when the emotional words are presented subliminally. An appropriate way to study early automatic processing is by using masked stimulation. Although automatic processes can be triggered by both conscious and unconscious stimuli, unconscious perception ensures automatic processing (Kiefer, 2007, 2012). In particular, unconscious emotional effects on language processing can be quite ubiquitous in everyday life experience, for example in social interactions, mass-media, marketing, political discourses and education, to name but a few. The interest of this approach thus seems undeniable. For this purpose, subliminal emotional adjectives (positive, negative, neutral) were inserted into neutral sentences just before the supraliminal sentential adjective, the latter being correct for half of the sentences and incorrect (number/gender disagreements between verbs and adjectives) for the other half. It has recently been demonstrated that masked words containing morphosyntactic anomalies with respect to conscious ongoing sentences can trigger syntactic processes (Jiménez-Ortega et al., 2014). These unconscious morphosyntactic anomalies also affected the syntactic processing of the conscious sentence. Accordingly, the impact of subliminal information on ongoing supraliminal syntactic processing has already been proved (see also Batterink and Neville, 2013, for evidence of unconscious syntactic processing).

In the present study, subliminal words did not contain morphosyntactic anomalies, but emotional information. In view of Jiménez-Ortega et al. (2014) and supraliminal studies with similar procedures (Martín-Loeches et al., 2012; Hinojosa et al., 2014), we expect that the syntactic processing of the sentence will be impacted by subliminal emotional words. The expected results are an increase in anterior negativity in response to morphosyntactic anomalies when the subliminal adjective is emotionally negative, as well as the absence of this component when the adjective is positive, as in Martín-Loeches et al. (2012) using supraliminal presentations. The results would contribute to recent—yet scarce—evidence supporting the impact of emotionally-laden language on syntax processes, while exploring whether the limits of these effects may surpass conscious presentations.

## Materials and Methods

### Participants

Twenty-four (out of an initial sample of 35; see below for details) Spanish-speaking volunteers participated in the experiment after giving informed consent according to the Declaration of Helsinki and with the approval of the ethics committee of the Hospital Clínico Universitario, UCM. This study was carried out in accordance with the recommendations of Hospital Clínico Universitario, UCM. The protocol was approved by the Hospital Clínico Universitario, UCM, Madrid, Spain. All of them were adults, with ages ranging from 18 to 51 (mean age = 28.7, SD = 9.8), self-reported normal or corrected-to-normal vision, and no history of neural or cognitive disorders, or reading difficulty. Half were female. All were right-handed, ranging from 10% to 100% (mean = 78%), according to the Edinburgh Handedness Inventory (Oldfield, 1971).

### Materials

We used 180 neutral Spanish sentences already used in previous experiments (Jiménez-Ortega et al., 2012, 2014; Martín-Loeches et al., 2012), which had been proved to be able to elicit both ANs and P600 components. The structure was: [determiner]-[noun]-[adjective]-[verb], as common in Spanish. Nevertheless, as used in a recent experiment (Jiménez-Ortega et al., 2014), we inserted another adjective, in this case subliminal to participants’ consciousness, starting 34 ms prior to the supraliminal adjective, lasting 17 ms, and followed by a hash mask also lasting 17 ms, preceding the supraliminal adjective. Thus, the complete structure was: [determiner]-[noun]-[subliminal adjective]-[mask]-[supraliminal adjective]-[verb], e.g., “El puente (alto; ####) romano permanece” [The (high; ####) Roman bridge stands]. This subliminal methodology was performed and evaluated in previous masked priming linguistic experiments (Van den Bussche et al., 2009; Kiefer et al., 2012; Jiménez-Ortega et al., 2014) with positive results. It should be noted that in Spanish, although uncommon, sentences with two adjectives are grammatically correct. Regardless of the uncommon structure, the design was common for all experimental conditions and therefore possible differences between conditions cannot be explained by structural features.

Accordingly, in addition to the 180 supraliminal adjectives, it was necessary to use three sets of 180 positive, neutral and negative subliminal adjectives. Therefore, for each supraliminal sentence we selected a matching positive, neutral and negative adjective (see Table 1 for examples). It was carefully controlled that all subliminal adjectives were semantically acceptable within the supraliminal sentence. The acceptability for each condition was calculated taking the number of results for the presence of a given noun followed by the adjective (e.g., “detective privado”) using Google. Thus, the average acceptability probabilities for the positive, neutral and negative conditions were 27882.3, 271209.5 and 15400.9 (SDs = 10889.15, 149942.8 and 6327.9, respectively). Therefore, the noun-adjective combinations used were quite common regardless of the condition. However, after Bonferroni correction, tendencies were observed for the neutral condition in comparison to the negative and positive ones (*t*_(358)_ = 2.17; *p* = 0.93 and *t*_(358)_ = 2.287; *p* = 0.69, respectively). No significant effects were observed between negative and positive conditions (*t*_(358)_ = 1.33; *p* > 0.05). This acceptability difference will be considered in the discussion section, in the light of the results obtained.

**Table 1:** **Types and examples of sentences used in the experimental procedure**.

		Determinant	Noun	Subliminal Adjectives: positive/neutral/negative	Mask	Supraliminal Adjectives: correct/incorrect	Verb
*Singular*	**Gender disagreement**	El	dinero	regalado contado falso	########	suelto suelta	tintinea
		*The*	*money*_[mas.]_	*gifted* *counted* *false*	*########*	*loose*_[mas.]_ *loose*_[fem.]_	*chinks*
	**Number disagreement**	La	norma	justa creada violada	########	escrita escritas	regula
		*The*	*rule*_[sing.]_	*fair* *created* *violated*	*########*	*written*_[sing.]_ *written*_[plural.]_	*regulates*
*Plural*	**Gender disagreement**	Los	muebles	arreglados barnizados desechos	#########	lijados lijadas	decoran
		*The*	*furniture*_[mas.]_	*repaired* *varnished* *damaged*	*########*	*sanded*_[mas.]_ *sanded*_[fem.]_	*decorate*
	**Number disagreement**	Las	frutas	sabrosas verdes descompuestas	########	maduras madura	abundan
		*The*	*fruits*_[plural.]_	*tasteful* *green* *rotten*	*########*	*ripen*_[plural.]_ *ripen*_[sing.]_	*abound*

*Literal translations (noun-adjective order inverted) into English, where mas., masculine; fem., feminine; sing., singular*.

Valence, arousal and frequency for positive, negative and neutral sets of adjectives (Table 2) were calculated according to published databases (frequency: RAE, 2016; valence and arousal: Stadthagen-Gonzalez et al., 2017). As expected, statistical analyses revealed significant effects for valence (One-way analysis of variance (ANOVA): *F*_(2,537)_ = 1157.4, *p* < 0.001; *post hoc*: all *t*s > 16.8, and *p*s < 0.001). Adjective frequency, length and percentage of participles were successfully controlled across conditions (all *F*s < 0.74, *p* > 0.05).

**Table 2:** **Means (and SDs) for linguistically-relevant variables in subliminal adjectives**.

	Valence	Arousal	Length	Frequency	Accep.*	Participles %
**Positive**	7.1 (0.75)	4.9 (0.8)	7.4 (1.6)	238.5 (271.6)	27882.3 (10889.15)	37.8
**Neutral**	5.7 (0.8)	4.9 (0.6)	7.5 (1.3)	244.2 (280.6)	271209.5 (149942.81)	30.6
**Negative**	3.1 (0.83)	6.3 (0.6)	7.4 (1.7)	254.8 (242.2)	15400.9 (6327.97)	37.8

*Acceptability of subliminal adjective supraliminal verbs combination.

Significant effects were also found for Arousal (*F*_(2,537)_ = 240.6, *p* < 0.001). *Post hoc* analyses revealed that whereas positive and neutral adjectives resembled each other in arousal (*t*_(358)_ = 0.34, *p* > 0.05), negative exhibited higher values when compared to both neutral and positive adjectives (*t*_(358)_ = 21.9, *p* < 0.01 and *t*_(358)_ = 17.5, *p* < 0.01, respectively). Due to the large number of adjectives needed and the fact that highly arousing negative adjectives were more frequent than positive and neutral ones in the largest and most recent Spanish database available (Stadthagen-Gonzalez et al., 2017), it was not possible to balance the mean arousal value of negative adjectives with positive and neutral ones without affecting other parameters such as frequency, word length, or semantic matching between subliminal adjectives and supraliminal sentences. We decided, therefore, to sacrifice arousal matching in order to control other variables, taking into consideration the results obtained by Espuny et al. (in preparation), in which explicit arousal manipulation did not affect syntactic processing. As in the acceptability case (see above), this circumstance will nevertheless be considered in the discussion section, in the light of the results obtained.

Though supraliminal adjectives were the same for all conditions, we also evaluated their valence, arousal, length and frequency, obtaining the following mean values, respectively: 5.1, 4.93, 6.63 and 273.9 (SDs = 1.1, 0.75, 1.2 and 301.5, respectively). As can be seen, these closely resemble those obtained for subliminal adjectives (with the exception of arousal for the negative ones). The supraliminal sentence could be either correct or incorrect, presenting in half of the cases a gender or a number disagreement in the adjective with respect to the preceding noun. Note that the subliminal adjectives were always syntactically correct relative to the supraliminal sentence.

All the combinations were distributed into six different sets, avoiding the repetition of any sentence within one set and assuring a counterbalance of undesirable variables such as frequency or word length, as well as of the different kinds of sentences. Each set contained 60 negative, 60 neutral and 60 positive subliminal stimuli evenly distributed in 180 trial sentences. Half of these sentences were syntactically correct and the other half included a supraliminal syntactic anomaly in the adjective. Therefore, subjects saw a given sentence only once, and in only one condition (emotion/correctness). In addition, each set contained 120 fillers resulting in 300 sentences per set (50% syntactically incorrect). Half of the fillers contained noun-adjective agreement violations. Filler sentences could be short (60 sentences: [determiner]-[subliminal noun]-[mask]-[supraliminal noun]-[verb]) or long (60 sentences: [determiner]-[noun]-[adjective]-[verb]-[subliminal complement]-[mask]-[supraliminal complement]), all of them previously tested and used in Jiménez-Ortega et al. (2012). The same filler sentences were included in each presentation set. Each complete set was presented to four participants, yielding the total sample of 24 considered in the analyses.

### Procedure

**Figure 1:**
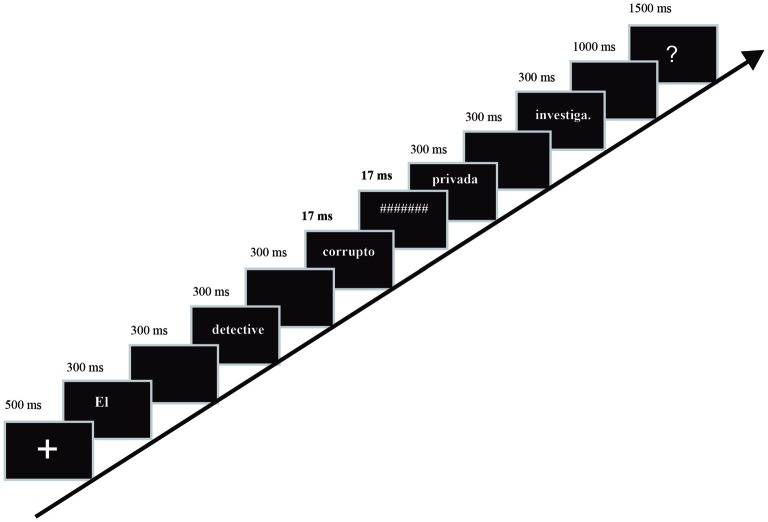
**Schematic representation of the stimulation procedure: emotional subliminal adjectives (positive, neutral, or negative) followed by a mask were presented during 17 ms (in bold) between the noun and the adjective of the supraliminal sentence, which could be correct or contain a number or gender disagreement**.

Participants were comfortably seated in a quiet shielded chamber, in front of an LCD screen (placed 65 cm from their eyes, visual angles around 0.8°–4° width) where the sentences were presented word-by-word in white letters against a black background in the center of the monitor. Each trial began with a fixation cross (500 ms) followed by the rest of the words one at a time (300 ms inter-stimulus-interval, 600 ms stimulus-onset-asynchrony, except for the subliminal adjective and the mask, whose exposition was adjusted to the screen refresh time, 17 ms). At the end of the sentence, after 1 s, a question mark was presented for 1.5 s, inducing the subject to respond regarding sentence acceptability. The inter-trial interval was 1 s. The first word of each sentence started with a capital letter, and all of the stimuli were presented using 30-point Arial font (Figure 1).

The participants were instructed to read the sentences and, when the question mark appeared, the subject had to indicate by pressing one of two buttons whether the sentence was syntactically correct or incorrect. The hand used for this task was counterbalanced across subjects. A set of 16 representative sentences, which were not included in the experiment, were provided for training. Participants were also asked not to blink between the fixation cross and the question mark during the electroencephalogram (EEG) recordings in order to avoid ocular artifacts.

After finishing the experiment, subjects were verbally asked about whether they had noticed something peculiar during the sentence presentation. Regardless of their answer, subjects thereafter performed a post-test, checking for the possible conscious perception of the subliminal stimuli. A sample of 48 sentences used in the previous experiment was presented here using the same procedures. After the presentation of each sentence, the following question was asked: “*Have you noticed something apart from the sentence?”* The possible answers were: “Yes” or “No”. If the answer was “No”, then the next sentence was presented. If the subjects answered, “Yes”, the following question was shown: “What did you notice?” Then, subjects could choose between two answers: “#####” or “*Other*”. Subjects were instructed to choose “*Other*” if they noticed an additional stimulus apart from the hash keys (and the supraliminal sentence). If they chose “*Other*”, they had to inform the experimenters verbally about it. Except for this last case, responses were given by pressing one of two keys, with the index and the middle fingers of the same hand. The presentation settings for the sentences were identical to those used during the experiment, except for answer delays and inter-trial intervals (for further details, see Jiménez-Ortega et al., 2014). Subjects were excluded from the data analyses in two cases: if they reported verbally that they were aware of subliminal presentation during the experiment, or if they simply detected more than six subliminal adjectives during the post-test. The exclusion of subjects was effected during data collection and before the data analyses, to keep sets balanced across participants.

#### Electrophysiological Recording

EEG was recorded according to the extended 10/20 International System (American Electroencephalographic Society, 1991; American Clinical Neurophysiology, 2006), by locating 27 tin electrodes embedded in an electrode cap (ElectroCap International) at the following locations: Fp1, Fp2, F7, F3, Fz, F4, F8, FC3, FC4, FT7, FT8, T7, C3, Cz, C4, T8, TP7, CP3, CP4, TP8, P7, P3, Pz, P4, P8, O1 and O2, and the right mastoid (M2). All of them were originally referenced to the left mastoid (M1) and later offline re-referenced to average mastoids (M1-M2). In order to control for ocular artifacts, VEOG and HEOG were also registered with electrodes above and below the left eye and at the outer canthus of each eye, respectively, for off-line eye-movement correction. A Brainamp^®^ amplifier was used, keeping electrode impedances below 3 kΩ. The signal was continuously recorded with a bandpass from 0.01 Hz to 100 Hz at a sampling rate of 250 Hz.

#### Data Analysis

The continuous recording of EEG was divided into time segments of 1100 ms, starting 200 ms previous to the onset of the subliminal adjective. ERP analyses were thus time-locked to subliminal adjectives instead of supraliminal ones, to avoid detrimental effects on baselines (supraliminal adjectives were presented 34 ms after subliminal ones). All EEG data were offline filtered with a band-pass filter of 0.01–30 Hz using Brain Vision Analyzer^®^. In addition, the method described by Gratton et al. (1983) was used to correct vertical (blinks) and horizontal eye movements. The artifacts were semi-automatically rejected offline, by eliminating epochs exceeding ± 100 μV in any of the channels. Any remaining epochs that contained artifacts were eliminated through visual inspection.

On average, for correct sentences, 22.5, 22.8 and 22.3 out of 30 trials (by subject) were included in the data analyses for positive, neutral and negative conditions, respectively, after removing epochs due to artifacts or incorrect responses. In contrast, for incorrect sentences, the averages for positive, neutral and negative conditions were 21, 20.3 and 21.3, respectively. A Correctness by Emotion ANOVA revealed significant effects for Correctness (*F*_(1,23)_ = 5.83, *p* < 0.05), but not for Emotion by Correctness interaction (*F*_(2,46)_ = 1.32, *p* > 0.1; *F*_(2,46)_ = 0.15, *p* > 0.1; respectively). Correctness effects were most probably a consequence of the increased number of errors observed for incorrect sentences (see data analyses below).

The next step was to perform repeated-measurement ANOVAs in which we contrasted clusters for six regions of interest (ROI): anterior, central and posterior regions, each one divided into two hemispheres. The Left Anterior cluster included Fp1, F7, F3 and FT7 electrodes; the Right Anterior cluster was composed by Fp2, F8, F4 and FT8; Left Central: T7, FC3, C3, CP3; Right Central: T8, FC4, C4, CP4; Left Posterior: TP7, P7, P3, O1; and Right Posterior: TP8, P8, P4, O2. We also included the Midline region: Fz, Cz and Pz. Thereafter, the ANOVAs included four factors: ROI (three levels: Anterior, Central, Posterior), Hemisphere (Left, Right), Correctness (Correct, Incorrect) and subliminal Emotion (Positive, Neutral, Negative). Violations of the sphericity assumption were corrected when necessary by the Greenhouse-Geisser method, and *post hoc* tests were corrected by the Bonferroni method. Time windows for measuring the syntactic ERP were selected after visual inspection of the waveforms.

## Results

A total of 35 subjects completed the experiment, although only 24 of them were considered in the data analyses. The remaining 11 subjects were excluded because at some point in the experiment they reported being aware of the emotional subliminal presentation. The post-test revealed that only 9 subjects out of the 24 that were finally included were aware of the subliminal word presentation at some point during this phase, a small proportion considering that they were asked specifically to pay attention to it. Even so, none of them could successfully identify more than 6 subliminal words out of 48.

The rate of exclusion was much higher than in a previous study with a similar subliminal presentation (Jiménez-Ortega et al., 2014), and is possibly the consequence of the fact that most of the subliminal words here are emotional, in contrast to the previous study, where all were neutral. To test this point, we analyzed the data for the subjects that detected subliminal adjectives (the 11 eliminated plus the nine participants that were aware of the subliminal word presentation at some point during the post-test), by means of a one-way ANOVA for Emotion factor. A substantial effect of Emotion was observed (*F*_(2,38)_ = 11.6, *p* < 0.001). The average numbers of adjectives detected were 8.8, 6.6 and 6.1 for positive, negative and neutral adjectives, respectively. *Post hoc* analyses revealed significances between positive and neutral and between positive and negative adjectives (*t*_(19)_= 5; *p* < 0.001 and *t*_(19)_ = 3.6; *p* < 0.01, respectively), though, significant differences were not observed between negative and neutral adjectives (*t*_(19)_= 0.75; *p* > 0.05).

### Behavioral Data

The total error rate for the sentence correctness task was 11.8%. The behavioral ANOVA analyses (including Correctness and Emotion factors) revealed a significant effect of Correctness (*F*_(1,23)_ = 5.63, *p* < 0.05). The percentage of errors for incorrect sentences (*M*s = 12.93) was larger than for correct sentences (*M*s = 9.63). However, neither the Emotion nor the Emotion by Correctness interaction yielded significant effects (*F*_(2,46)_ = 1.35, *p* > 0.1; *F*_(2,46)_ = 0.77, *p* > 0.1; respectively).

A effect of Correctness was also observed for reaction times (*F*_(1,23)_ = 5.2, *p* < 0.05), these being longer for correct conditions than for incorrect ones (*M*s = 439.69 vs. 412.73 ms). As for error rates, neither Emotion nor Emotion by Correctness interactions yielded significant effects (*F*_(2,46)_ = 0.76, *p* > 0.1; *F*_(2,46)_ = 0.43, *p* > 0.1; respectively).

### ERP Data

**Figure 2:**
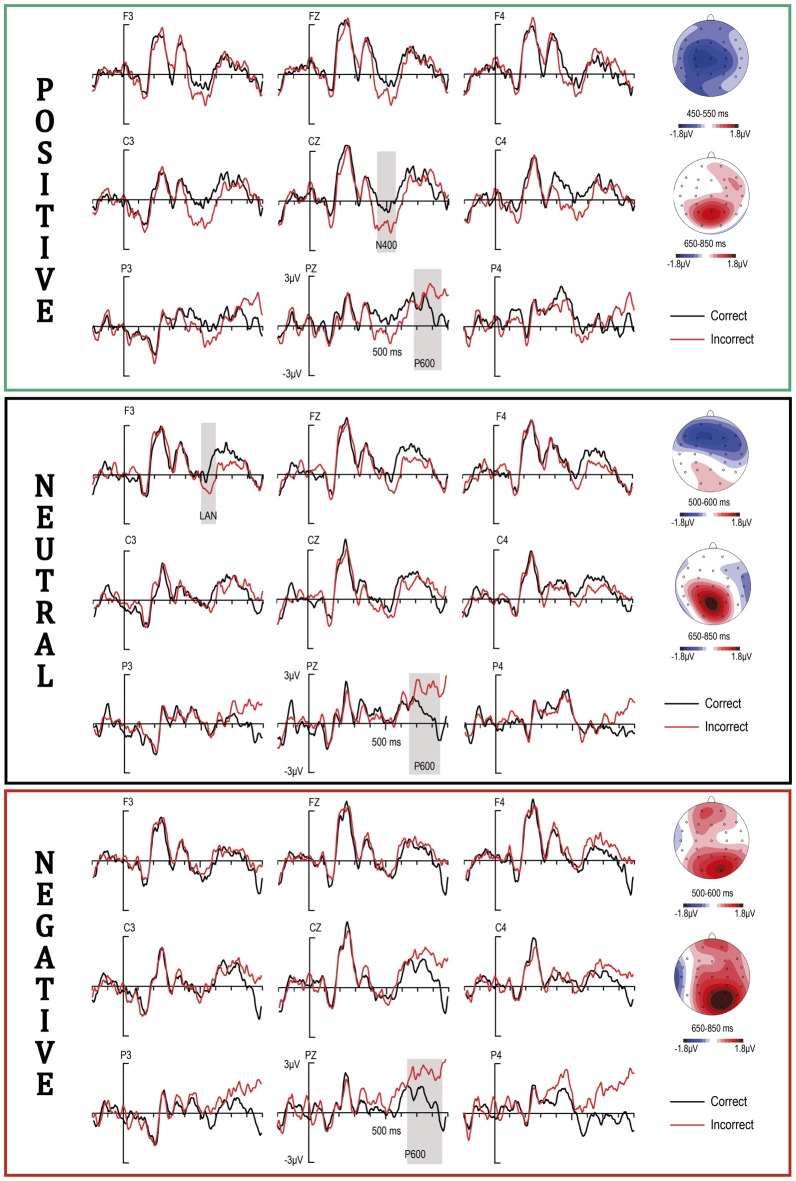
**Event-related brain potential (ERP) response to syntactically correct and incorrect supraliminal adjectives for positive, neutral and negative conditions**.

**Figure 3:**
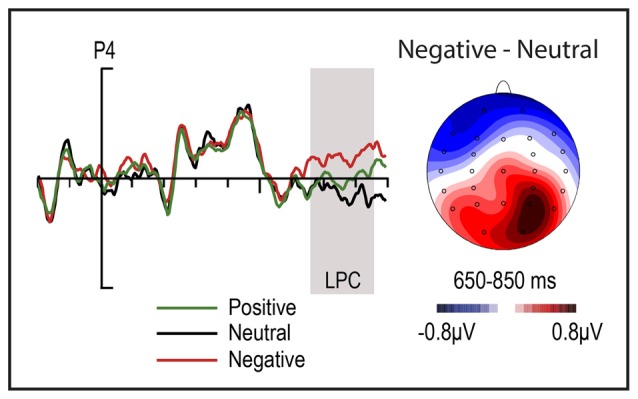
**Emotional effects of positive, negative and neutral subliminal adjectives regardless of correctness factor**.

Overall, visual inspection of the ERPs (Figure 2) revealed an anterior negativity to supraliminal grammatical violations for subliminal neutral condition, but not for the other conditions. Further, instead of an anterior negativity, an N400 component seemed to appear for the positive condition. A P600 was also observed for all three emotional conditions. Finally, a negativity around 400 ms and a late emotional effect (LPC) were also observed, regardless of correctness, for the negative condition (Figure 3).

#### Correctness by Subliminal Emotion Interactions (450–550 ms)

Visual inspections indicated that the positive condition might be better characterized as displaying an N400 for morphosyntactic violations, in this case peaking in the 450–550 ms time range. The general ANOVA in this window showed a trend for the Emotion by Correctness interaction (*F*_(2,46)_ = 3.29, *p* = 0.07). However, *post hoc* analyses consisting of an ROI by Hemisphere by Correctness ANOVA within each emotional condition separately supported our visual impression. In this regard, in the positive condition alone, these analyses yielded a significant effect of Correctness (*F*_(1,23)_ = 5.17, *p* < 0.05), whereas in the neutral and the negative conditions no significant effects emerged (*all F*s < 1.6). Overall, a widespread N400 maximal around the central regions (see the map in Figure 2) seems to be supported for the positive condition in the 450–550 ms time range, while no relevant effects emerged for negative and neutral conditions.

#### Correctness by Subliminal Emotion Interactions (500–600 ms)

In order to analyze the anterior negativity apparent in Figure 2 for morphosyntactic violations in the neutral condition, a window was established for ANOVA analyses in the 500–600 time range. This revealed significant effects for Hemisphere by ROI by Correctness by Emotion interaction (*F*_(4,92)_ = 3.91, *p* < 0.05) and trends for Correctness by Emotion, as well as for ROI by Emotion interaction (*F*_(4,92)_ = 2.6, *p* = 0.088; *F*_(2,46)_ = 2.82, *p* = 0.07; respectively). No significant effects were observed for all the other factors or interactions (*all F*s < 1.9). Further, ROI (3) by Correctness (2) ANOVA analyses within positive, neutral and negative conditions separately, were calculated to check for correctness effects according to the main aim of this study. Neutral condition analyses yielded Correctness factor effects and also Hemisphere by ROI by Correctness interaction significances (*F*_(1,23)_ = 6.87, *p* < 0.05; (*F*_(2,46)_ = 9.01, *p* < 0.01, respectively). For the negative condition, significances were observed for ROI by correctness, and Hemisphere by ROI by correctness interactions (*F*_(2,46)_ = 9.15, *p* < 0.01; *F*_(2,46)_ = 8.7, *p* < 0.01, respectively). In contrast, neither factor nor interaction significances were obtained for the positive condition (*all F*s < 1.41). *Post hoc* analyses revealed that significant ROIs were different for the negative condition in comparison to the neutral one. For the negative condition, the Correctness factor was significant for posterior and central regions (*F*_(1,23)_ = 4.93, *p* < 0.05; *F*_(1,23)_ = 7.43, *p* < 0.01, respectively), while for the neutral condition significances were observed for the left anterior region (*F*_(1,23)_ = 4.07, *p* < 0.05). None of the other *t*-test comparisons recorded significant effects (*all F*s < 1.7, *p*s > 0.1).

Overall, the data in the 500–600 ms time range support a significant anterior negativity for morphosyntactic violations in the neutral condition, as well as a posterior positivity in the negative condition that might be interpreted as reflecting an earlier onset of the P600 component that will peak later on (see below). Similarly, both visual inspection and data analyses (ROI by Emotion trend, reported above) point to an onset of emotional modulations peaking later (see below). Finally, no relevant effects were supported for this time range in the positive condition.

#### Emotion and Correctness: Main Effects (650–850 ms)

On the one hand, visual inspections pointed to the existence of an LPC component at least when comparing negative and neutral emotions. On the other hand, visual inspection of the P600 (Figure 2) suggests P600 amplitude differences between emotions, with the lowest values in the positive one. However, the main analyses yielded significances for ROI by Emotion interaction (*F*_(2,46)_ = 3.47, *p* < 0.05) and also for Hemisphere by ROI by Correction (*F*_(2,46)_ = 4.09, *p* < 0.05), but neither trends nor significances were obtained for any other interaction (*all Fs* < 2.11; *p* < 0.1). Therefore, in the latter case statistical analyses discard the emotional effects on the P600 component indicated by the visual inspections. Since the main aim of this article is to investigate subliminal emotional effects on syntax, further *post hoc* analyses for correctness are not included here, for simplicity (Figure 2).

To further test the emotional effects reported above, we calculated three 3 × 2 ANOVA analyses (ROI by Emotion) comparing emotions pair-wise. Significant ROI by Emotion interactions were observed when comparing negative and neutral emotions (*F*_(2,46)_ = 4.9, *p* < 0.05), and a trend between negative and positive emotions (*F*_(2,46)_ = 3.38 *p* = 0.07). No significant effects were observed between positive and neutral conditions (*F*_(2,46)_ = 1.25 *p* > 0.1; Figure 3). In addition to these emotional effects, visual inspections also pointed to a previous emotional effect: between 400 ms and 500 ms the negative condition showed a frontal negativity in comparison to neutral and positive ones. However, neither Emotion nor other main factors and interactions reached significant levels (*all Fs* < 1.4; *p*s > 0.1; Figure 3).

## Discussion

In this study, we aimed to explore whether and how morphosyntactic processing might be modulated by subliminal presentations of emotional adjectives appearing just prior to the presentation of sentential supraliminal adjectives, including gender or number agreement violations. The results were positive in the sense that significant modulations were observed, both at the ERP as well as at the behavioral levels.

The most immediate observation is that 11 out of 35 subjects had to be excluded from the data analyses because at some point they were aware of the emotional subliminal presentation. Using a very similar methodology, but presenting neutral subliminal adjectives, none of the subjects were excluded in a previous study (Jiménez-Ortega et al., 2014). A major explanatory factor could therefore be the emotional nature of the subliminal adjectives in the present study, in consonance with recent studies in which enhanced detection is reported for emotional stimuli in attentional blink paradigms (Kanske et al., 2013). Our data support this possibility, although in our case the effect seems mainly supported by positive adjectives, since the analyses revealed that these adjectives were significantly easier to detect (see “Results” Section for details).

Behavioral data revealed that the participants showed more problems in identifying incorrect sentences as against correct sentences as measured by error rates. This is in contradiction with previous studies using similar sentences (Jiménez-Ortega et al., 2012; Martín-Loeches et al., 2012) and is probably a consequence of the linguistic processing of the subliminal adjective. The latter was always correct with respect to the on-going supraliminal sentence, hence yielding a conflict when the supraliminal adjective was incorrect, this increasing error rates. In Jiménez-Ortega et al. (2014), with a very similar procedure, this type of conflict might also have existed, but error rates did not show this atypical result. However, in that study subliminal adjectives presented two main differences from the present study. First, they could be either correct or incorrect, minimizing the overall strength of possible sub- vs. supraliminal conflicts. Second, in Jiménez-Ortega et al. (2014), the subliminal adjectives were always emotionally neutral, while two thirds in the present study were emotionally valenced. The overall saliency of the subliminal adjectives used here would therefore have been higher than in our previous study, hence increasing the strength of subliminal vs. supraliminal conflicts.

In line with these interpretations it might be the appearance of a LAN in response to morphosyntactic violations in the neutral condition with a noticeably late latency. Typically, LANs peak around 300–500 ms, whereas here it was between 550 ms and 750 ms. The conflict between a subliminal correct adjective and a supraliminal incorrect one might be the basis for this noticeable delay. Although in the neutral condition the subliminal adjective, by definition, was not emotional, the majority of emotional subliminal adjectives might again have influenced such an effect.

Also of interest were the ERP results for the negative and positive conditions, where no frontal negativities were observed. While the positive condition seemed to yield an N400 component though this result should be considered with caution, see below-, no negativity but an earlier onset of the P600 component appeared in the negative condition. A possible explanation for the latter results might be that in negative adjectives arousal levels were larger than those for positive and neutral ones (see “Materials and Methods” Section) this being a limitation of the present study. In addition, the acceptability for subliminal adjectives and supraliminal verb combinations was higher for neutral than negative and positive conditions. This may contribute to an amplitude increase of positive N400, but it neither explains the lack of an early component for negative subliminal adjectives, nor the lack of differences in late components among conditions (For cloze probability modulations see, DeLong et al., 2014). However, although these limitations cannot be disregarded for the present data, an alternative explanation based on valence-specific information can also be considered. In a previous study, the authors (Martín-Loeches et al., 2012) reported a significant increase of the LAN component for morphosyntactic violations in negatively valenced adjectives. This is contrary to the present results, in which a subliminal negative adjective preceding the violation seemed to eliminate ANs. In Martín-Loeches et al. (2012), the presence of a morphosyntactic violation and a negative valence occurred simultaneously, i.e., in the same word. Considering a possible “negativity bias” for a prevailing summoning of resources by the negative valence information (Carretié et al., 2009), the increase in anterior negativity was interpreted there as a result of greater efforts for early syntactic processing. The negative valence in the present study, however, occurred slightly before the actual occurrence of the morphosyntactic violation, even if subliminally. This time preeminence in conjunction with a presumed negativity bias might be sufficient for negative, morphosyntactically correct adjectives to capture early and automatic syntactic resources (Jiménez-Ortega et al., 2014). This, in turn, would have been detrimental for subsequently processing supraliminal morphosyntactic violation. As a consequence, no anterior negativity would emerge under these conditions. This interpretation is in line with Ding et al. (2016) with morphosyntactic anomalies preceded by emotional words.

The absence of the LAN response to morphosyntactic violations in the positive condition nevertheless parallels our previous finding for positive adjectives with morphosyntactic violations (Martín-Loeches et al., 2012). On that occasion, the data suggested that positive words within a sentence are not parsed in a first-pass in the same way as neutral or negative words. Positive words actually seemed to induce heuristic processing strategies (Holt et al., 2009). This is in line with studies reporting that positive emotional states increase the use of heuristic strategies, less computationally demanding than algorithmic ones (e.g., Blanchette and Richards, 2010). As suggested in our previous study, it is possible that, at the cognitive level at least, some of the strategies elicited by positive emotional states might be triggered when a positive word appears in a sentence. If the relative induction of a heuristic processing style by subliminal positive words is assumed, the presence of the N400 semantic component elicited here by morphosyntactic violations following a just-presented subliminal positive adjective would therefore not be totally unexpected. On the one hand, semantic processing is often considered a heuristic process (Vissers et al., 2007; Berkum et al., 2009; Martín-Loeches et al., 2009). On the other hand, N400 instead of ANs in response to agreement anomalies has been previously reported (e.g., Barber and Carreiras, 2003, 2005; Wicha et al., 2004; Molinaro et al., 2008; Mancini et al., 2011), to such an extent that some authors consider that AN and N400 are not categorically distinct ERP components (for a detailed discussion, Molinaro et al., 2015). It has been rather interpreted as reflecting the use of alternative strategies to solve morphosyntactic violations, such as the use of discourse levels of analysis to judge material correctness. That is, N400 components would appear when lexico-semantic information processing is required (Molinaro et al., 2011, 2015). Nonetheless, a cautionary note should be struck here, as our N400 response to positive subliminal adjectives was supported statistically by *post hoc* analyses only, the overall ANOVA analyses yielding a trend for significance. Nonetheless, though not sufficiently robust in statistical terms, the result appears to us openly assumable, particularly considering its consonance with previous findings and interpretations, as outlined here.

Although the P600 component appeared earlier in the negative condition, this is probably due to a lack of LAN or N400 components in this condition, as already mentioned. In fact, the P600 did not significantly differ at its peak between emotional conditions. This is in line with Van Berkum et al. (2013), where the P600 component was not affected by mood induction using sad and happy films, except for a slightly earlier onset of P600 in the happy mood condition. This is also in line with previous studies in which P600 does not seem to be significantly affected by emotional information (Jiménez-Ortega et al., 2012; Martín-Loeches et al., 2012). However, several studies have reported a reduction in the P600 component for sad as compared to induced happy mood (Vissers et al., 2010; Verhees et al., 2015). Nevertheless, these latter studies did not report traces of ANs or N400 components and, overall, methodological differences may account for these discrepancies. More research seems needed to better clarify the effects of emotional information on the P600 syntactic component.

ERP data showed a late effect of emotion (LPC) independent of sentence correctness; negative subliminal adjectives elicited increased LPC amplitudes in parietal electrodes as compared to both neutral and positive subliminal adjectives (Figure 3). Some studies presenting conscious emotional words reported similar, or larger, LPC amplitudes for positive in comparison to negative conscious emotional words (Kissler et al., 2007; Schacht and Sommer, 2009a), while other authors, in line with our results, reported the opposite effects (Gootjes et al., 2011; Imbir et al., 2015). In our case, however, the larger LPC for neutral and negative might be a consequence of the higher arousal value of negative adjectives in comparison to neutral and negative subliminal adjectives. In any event, the presence of these modulations in response to our subliminal stimuli vouches for the efficacy of our procedures in eliciting subliminal word processing and the intended emotional effects beyond the data discussed above on morphosyntactic processing.

Given that ANs reflect early automatic stages of language comprehension (Hasting and Kotz, 2008; Batterink and Neville, 2013; Jiménez-Ortega et al., 2014; Lucchese et al., 2017) and that this early automatic processing seems to be affected by unconscious emotional information, our results fully support interactive models of language, that is, interactions between lexico-semantic and syntactic domains even at early stages (e.g., MacDonald et al., 1994; Novick et al., 2003; Kuperberg, 2007; Pickering and Garrod, 2013). Our results also add to growing evidence supporting a high overlap and interdigitation between emotion and cognition networks in the brain (Pessoa, 2008, 2012, 2015a,b, 2016).

Finally, it has been demonstrated here that emotional information of which the reader is unaware can interact with syntactic processing of sentences at early stages. The relevance of findings like these should therefore be taken into consideration, not only for their contribution to language models, but also for countless daily-life situations and contexts in which the comprehension of linguistic messages is crucial, as the latter appears vulnerable to unnoticed information in our surroundings.

## Author Contributions

LJ-O and MM-L: experiment design; CV-R, LJ-O and PHT: linguistic material elaboration; LJ-O and JE: programing; CV-R, JE, PHT and LJ-O: data collections; LJ-O: data analysis; MM-L, LJ-O and JE: data interpretation; LJ-O and MM-L: manuscript writing; JE, LJ-O and MM-L: manuscript correction.

## Conflict of Interest Statement

The authors declare that the research was conducted in the absence of any commercial or financial relationships that could be construed as a potential conflict of interest.
